# Sunday Driver Mediates Multi-Compartment Golgi Outposts Defects Induced by Amyloid Precursor Protein

**DOI:** 10.3389/fnins.2021.673684

**Published:** 2021-06-01

**Authors:** Qianqian Du, Jin Chang, Guo Cheng, Yinyin Zhao, Wei Zhou

**Affiliations:** ^1^Britton Chance Center for Biomedical Photonics, Wuhan National Laboratory for Optoelectronics-Huazhong University of Science and Technology, Wuhan, China; ^2^MoE Key Laboratory for Biomedical Photonics, Collaborative Innovation Center for Biomedical Engineering, School of Engineering Sciences, Huazhong University of Science and Technology, Wuhan, China

**Keywords:** amyloid precursor protein, Golgi outposts, motor protein, Sunday driver, dendrite defects

## Abstract

Golgi defects including Golgi fragmentation are pathological features of Alzheimer’s disease (AD). As a pathogenic factor in AD, amyloid precursor protein (APP) induces Golgi fragmentation in the soma. However, how APP regulates Golgi outposts (GOs) in dendrites remains unclear. Given that APP resides in and affects the movements of GOs, and in particular, reverses the distribution of multi-compartment GOs (mcGOs), we investigated the regulatory mechanism of mcGO movements in the *Drosophila* larvae. Knockdown experiments showed that the bidirectional mcGO movements were cooperatively controlled by the dynein heavy chain (Dhc) and kinesin heavy chain subunits. Notably, only Dhc mediated APP’s regulation of mcGO movements. Furthermore, by loss-of-function screening, the adaptor protein Sunday driver (Syd) was identified to mediate the APP-induced alteration of the direction of mcGO movements and dendritic defects. Collectively, by elucidating a model of bidirectional mcGO movements, we revealed the mechanism by which APP regulates the direction of mcGO movements. Our study therefore provides new insights into AD pathogenesis.

## Introduction

The Golgi apparatus, an organelle with a unique cisternal stacked structure, is responsible for protein trafficking, sorting, and processing. Accumulated evidence indicates that there are defects in the structure and function of the Golgi apparatus in brains affected by Alzheimer’s disease (AD). For example, fragmentation and atrophy of the Golgi apparatus have been observed in AD neurons (Stieber et al., 1996; Baloyannis and Ma, 2000; Baloyannis, 2014). Abnormalities of protein glycosylation have also been reported in AD brain tissues, such as an enhancement of glycosylation of the Tyr residue of β-amyloid (Aβ) peptides (Halim et al., 2011) and a decrease of neural sialyltransferase activity (Maguire and Breen, 1995). These results suggest that defects in the Golgi apparatus are pathological features of AD.

Previous studies have suggested that defects in the Golgi apparatus are induced by the amyloid precursor protein (APP). APP, the precursor of Aβ, is one of the key pathogenic factors in AD (Golde et al., 1992; Hardy and Higgins, 1992; Sisodia and Price, 1995). Studies have shown that overexpressing APP in neurons leads to Golgi fragmentation, accompanied by swollen cisternae and disorganized stacks (Joshi et al., 2014). The mechanism of Golgi fragmentation has been elucidated: the accumulation of Aβ produced by amyloidogenic APP processing induces the phosphorylation of Golgi structural proteins, such as GRASP65 (Joshi et al., 2014) and GM130 (Sun et al., 2008). Hence, we raise the hypothesis that overexpression of APP leads to Golgi defects, then induces the degeneration of the structure and function of neurons, thereby accelerating AD development.

In neurons, Golgi apparatus exists not only in the soma but also in the dendrites, where they are known as Golgi outposts (GOs) (Pierce et al., 2001; Horton and Ehlers, 2003; Horton et al., 2005). Compared to somal Golgi apparatus, dendritic GOs have their own unique structure and function. GOs have a different compartmental organization, including single-compartment GOs (scGOs) and multi-compartment GOs (mcGOs), with disconnected *cis-*, *medial*-, and *trans*-cisternae in the former and stacked in the latter (Zhou et al., 2014). Meanwhile, different from the somal Golgi apparatus with a stationary positioning, GOs are highly mobile, traveling in an anterograde (toward the dendritic ends) or retrograde direction (toward the soma) (Horton and Ehlers, 2003; Ye et al., 2007). The dynamic GOs are involved in the local processing and transport of proteins (Horton and Ehlers, 2003; Valenzuela and Perez, 2015), for example, the NMDA receptors (NMDARs) are selectively transported to synapses *via* GOs while bypassing the Golgi apparatus (Jeyifous et al., 2009). Stationary GOs have also been found to be involved in the growth and stability of dendritic branches by functioning as local acentrosomal microtubule nucleation sites (Ori-McKenney et al., 2012). Alterations of GOs have been reported in neurodegenerative diseases: for example, a loss of GOs has been seen in models of Machado-Joseph’s disease (Chung et al., 2017) and amyotrophic lateral sclerosis (Park et al., 2020), and abnormal GO dynamics are induced by leucine-rich repeat kinase (Lrrk), a Parkinson’s disease-associated protein (Lin et al., 2015). However, the mechanism by which APP regulates GOs remains unknown.

Here, inspired by the finding that APP alters the distribution of mcGOs, we investigated the molecular mechanism by which APP regulates the direction of mcGO movements. First, *in vivo* imaging showed the colocalization between APP and GOs, and further revealed the dynamics and distribution of GOs. Then, by the knockdown of motor protein subunits, we investigated the mechanism that determines the direction of mcGO movements. This allowed us to determine the subunit that mediates the alteration of the direction of mcGO movements induced by APP. Finally, by loss-of-function screening, we discovered the adaptor protein that is specifically involved in APP’s regulation of the direction of mcGO movements, and its rescue of dendritic morphology was explored.

## Materials and Methods

### Fly Stocks and Transgenic Line

The stocks of flies published in this study included: GAL4^19– 12^ (Xiang et al., 2010); UAS-GalT:TagRFPt, UAS-ManII:TagRFPt/GFP (Ye et al., 2007; Zhou et al., 2014); UAS-CD8:GFP (Lee and Luo, 2001); UAS-APP:GFP^*CG*^, UAS-APP695^*N–myc*^, and UAS-APP695-Swedish^*N–myc*^ (a gift from Lei Xue, Tongji University, China); the transgenic fly stocks for the motor proteins subunits and adaptor proteins were shown in Supplementary Tables 1, 2, respectively. To generate the UAS-Syd:EGFP transgenic line, the *Sunday driver* (*Syd*) cDNA (#RE24340, BDGP) and *EGFP* cDNA were amplified by PCR and transferred into the vector pJFRC2-10 × UAS-IVS-mCD8-GFP (plasmid #: 26214, Addgene, Cambridge, MA, United States). The construct was then injected into embryos of PBac{y[+]-attP-3B}VK00033 to generate transgenic flies. All stocks and crosses were maintained in a 25°C incubator with 40–60% humidity.

### Dissection, Immunostaining, and Imaging

Third instar larvae were dissected in insect saline and fixed with 4% formaldehyde (PFA) for 40 min; permeabilized and washed in a wash buffer (1X PBS containing 0.15% Triton X-100), and then blocked in 5% normal serum for 30 min. After washing, larval filets were incubated overnight at 4°C with the primary antibody and 3 h with the second antibody at room temperature, avoiding light. The antibodies included: chicken anti-GFP (1:5,000, Aves Labs, Inc., Tigard, OR, United States); Alexa647 anti-HRP (1:1,000; Jackson ImmunoResearch Labs Inc., West Grove, PA, United States); and Alexa488 anti-chicken (1:250; Jackson ImmunoResearch Labs Inc., West Grove, PA, United States). The larval filets were then dehydrated by alcohol and transparentized with xylene before mounting on the slide with DPX (Sigma-Aldrich, St. Louis, MO, United States) for imaging. The C3da neurons in the dorsal cluster of the fourth to sixth abdominal segments of the larvae were imaged using a confocal microscope (FV1000, Olympus, Japan) under a 60x oil immersion objective (NA = 1.42, Olympus, Japan). The images were captured at a resolution of 1,024 × 1,024 pixels and a z step of 0.4 μm in XYZ mode with sequential scanning. Images of whole neurons require multiple views to be spliced together.

### Morphological Analysis

For dendritic morphology analyses, we manually traced the dendritic branches by the NeuronJ plugin (Meijering et al., 2004). The tracings were used to measure the dendrite length and count the number of dendritic branch points. The number and length of spikes were similarly obtained.

To quantify the colocalization between Golgi and APP (or Syd), we calculated the proportion of colocalized puncta. To count the number of total Golgi and total APP (or Syd) as well as the colocalized ones, the Golgi and APP puncta were separately cataloged in ROIs manager, and the puncta that overlapped with the puncta on the other channel were counted as a colocalization (as shown in yellow). For the purposes of analyzing the compartmental organization of GOs, we classified the colocalized GalT:TagRFPt and ManII:GFP puncta as mcGOs, while others as scGOs.

To evaluate the specificity of *Syd* RNA-interference (RNAi), we examined the expression level of Syd by the fluorescence of Syd-GFP. For this, the soma of C3da neurons were outlined for the ROIs, then the average fluorescence in the ROIs was measured automatically.

### *In vivo* Imaging

*In vivo* imaging was performed as previously described (Zhou et al., 2014). Firstly, the third instar larvae were collected and anesthetized with ether. The orientation of larvae was adjusted until the ventral trachea was located in the middle of the view field, and then, the larvae were mounted in a drop of halocarbon oil. The movements of larvae were further limited by pressing a coverslip mounted on top of a vacuum grease. Time-lapse imaging of fluorescently tagged GOs in C3da neurons was acquired using a confocal microscope (FV1000, Olympus, Japan) under a 60x oil immersion objective. The settings for the two channels were: APP/ManII (488 nm laser for GFP, emission filter: 500–525 nm) and GalT (543 nm laser for RFP, emission filter: BA560IF). Multichannel time-lapse images were captured at zoom 2 with a resolution of 512 × 512 pixels and collected in the XYZT mode for 10 min at 6 s intervals.

### Quantification of GO Dynamics

To quantify the dynamic behaviors of GOs, we generated kymograph images (as X-T images shown) using ImageJ. The kymograph images were generated in four steps: stabilization, straightening, reslicing, and timing projection. First, images were stabilized using the Stabilizer plugin (Li, 2008) to avoid fluctuation from larvae movements. We straightened the dendrites to accurately measure the distances of GO movements in the dendrites. The dendrites beyond the first branch points were traced and straightened over the entire stack. The straightened images were resliced to reconstruct the corresponding slices across the time series. Finally, the resliced images were time projected to produce a kymograph of GOs in the dendrites.

Furthermore, we determined the number of dynamic GOs and analyzed their properties, including direction and displacements. We compared the position of GOs at the start and end of the movement, and classified the movement as anterograde when the final position of GOs puncta relative to the starting point was toward the dendritic ends, and retrograde when the final position of GOs was toward the soma. The coordinates of GOs trajectories on the kymograph were measured to obtain the displacement information during movements. Meanwhile, the mcGOs and scGOs were distinguished based on the colocalization analysis for ManII and GalT. Finally, the GOs trajectories were traced, and combined tracks of GOs were generated by aligning the starting points of the tracings, as shown in Figures 2C, 3C, 4D. Besides, we defined the difference between dynamic and stationary GOs and classified them as stationary if they moved less than 0.5 μm in any direction, otherwise as dynamic. To circumvent the influence of the physiological state of neurons and the accuracy of calculation, only the kymograph images with at least one moving GOs were analyzed.

**FIGURE 1:**
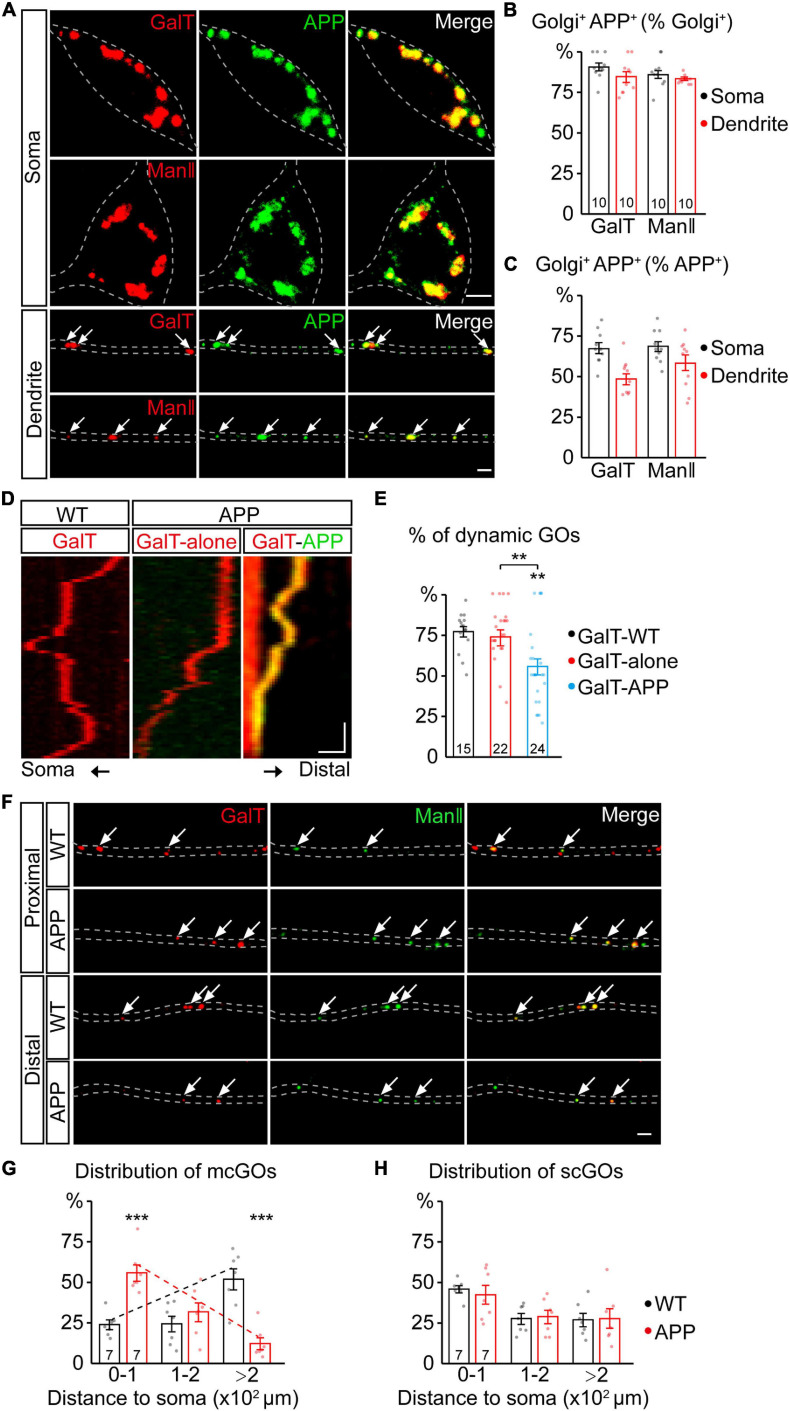
APP reverses the distribution of multi-compartment Golgi outposts (mcGOs) in the dendrites. **(A–C)** APP resides in the vast majority of GOs. **(A)** Examples of APP (green) and Golgi complex (red) in the soma and dendrites. APP is labeled by APP-GFP and Golgi complex is labeled by GalT-TagRFPt (top, *trans*-Golgi) or ManII-TagRFPt (bottom, *medial*-Golgi). Arrows indicate colocalized APP and GOs in the dendrites. **(B,C)** Quantification of the proportion of colocalized APP and Golgi complex in the soma and dendrites: co-localization as a percentage of Golgi complex **(B)** or APP **(C)**. **(D,E)** The resident APP changes the dynamics of GOs. **(D)** Examples of dynamic GOs with and without APP. Kymograph of time-lapse imaging of GOs showing GO (labeled by GalT-TagRFPt) dynamics without (red) or with APP-GFP (yellow, GalT-APP). Scale bar: 2 μm/1 min. **(E)** Bar charts showing the proportion of dynamic GOs with or without APP. **(F–H)** APP reverses the distribution of mcGOs in the dendrites but not that of single-compartment outposts (scGOs). **(F)** Examples of the distribution of GOs in the wild-type and APP neurons. The *medial*- and *trans*-Golgi are labeled by ManII-GFP (green) and GalT-TagRFPt (red), respectively. Arrows point to mcGOs. **(G,H)** the distribution patterns of **(G)** mcGOs and **(H)** scGOs in dendrites. Scale bar: 2 μm. Statistical significance was assessed with ANOVA tests in **(E)**, and Student’s *t*-test in **(G,H)** (***P < 0.001; **P < 0.01).

**FIGURE 2:**
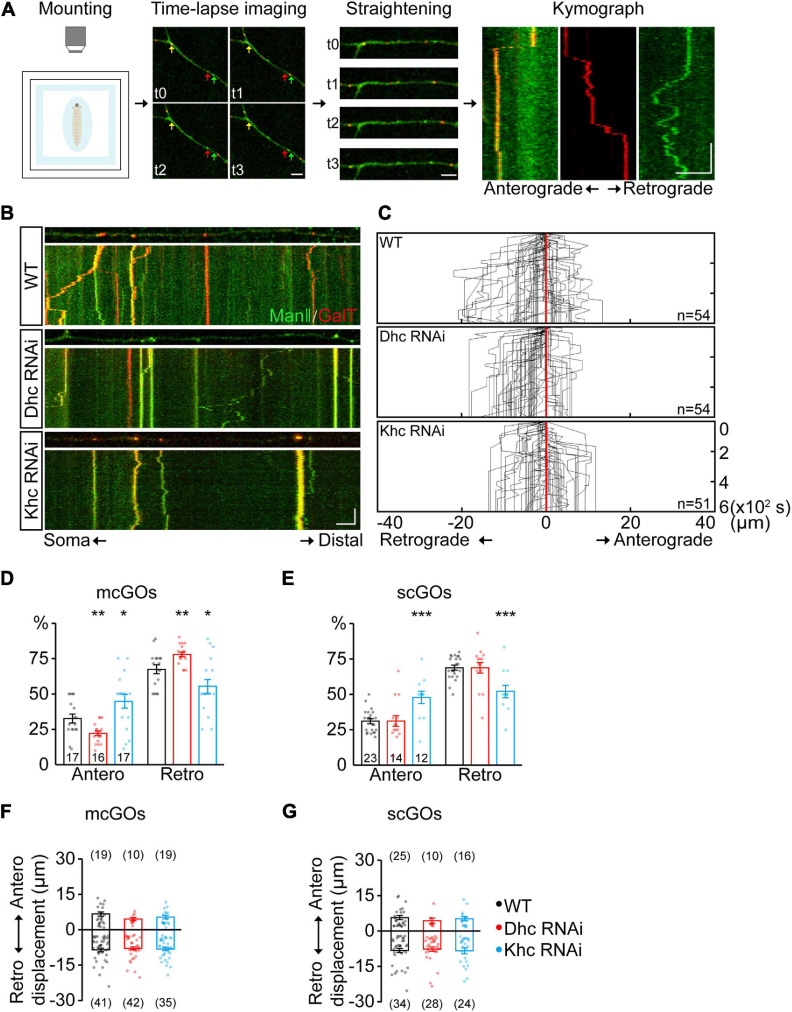
Dhc and Khc coordinate to maintain the equilibrium of anterograde/retrograde mcGO movement. **(A)** Schematic diagram showing the pipeline for the generation of GO trajectories. The four steps are (from left to right): the anesthetized larvae were (1) mounted for subsequent (2) time-lapse imaging. Arrows with different colors in the image indicate the GOs with a different organization. (3) The dynamic puncta in the dendrites of consecutive images were tracked down by the segmented line tool and (4) processed to generate a kymograph. **(B)** Examples of the trajectories of dynamic GOs in the wild-type and APP neurons with RNAi knockdown of dynein heavy chain (*Dhc*) or kinesin heavy chain (*Khc*). *Top*: snapshots of the GOs in the straightened dendrites. *Bottom*: kymograph of time-lapse imaging of GOs for 10 min. Dual-color live imaging showing the compartmental organization of GOs in dendrites, which are labeled by ManII-GFP (green, *medial*-Golgi) and GalT-TagRFPt (red, *trans*-Golgi). Green or red indicates a scGO and yellow indicates the mcGO. Scale bar: 5 μm/2 min. **(C)** Compound tracks of dynamic GOs. Red lines are aligned along with displacement 0; puncta numbers are indicated within the boxes. **(D,E)** Quantification of the percentage of anterograde and retrograde movements for **(D)** mcGOs and **(E)** scGOs. The percentage of anterograde (retrograde) movements is calculated by dividing the number of anterograde- (retrograde-) moving mcGOs by the total number of moving mcGOs. **(F,G)** Quantification of the displacements of **(F)** mcGOs and **(G)** scGOs. Anterograde displacements are shown as positive and retrograde as negative. Statistical significance was assessed with Student’s *t*-test (***P < 0.001; **P < 0.01; *P < 0.05).

**FIGURE 3:**
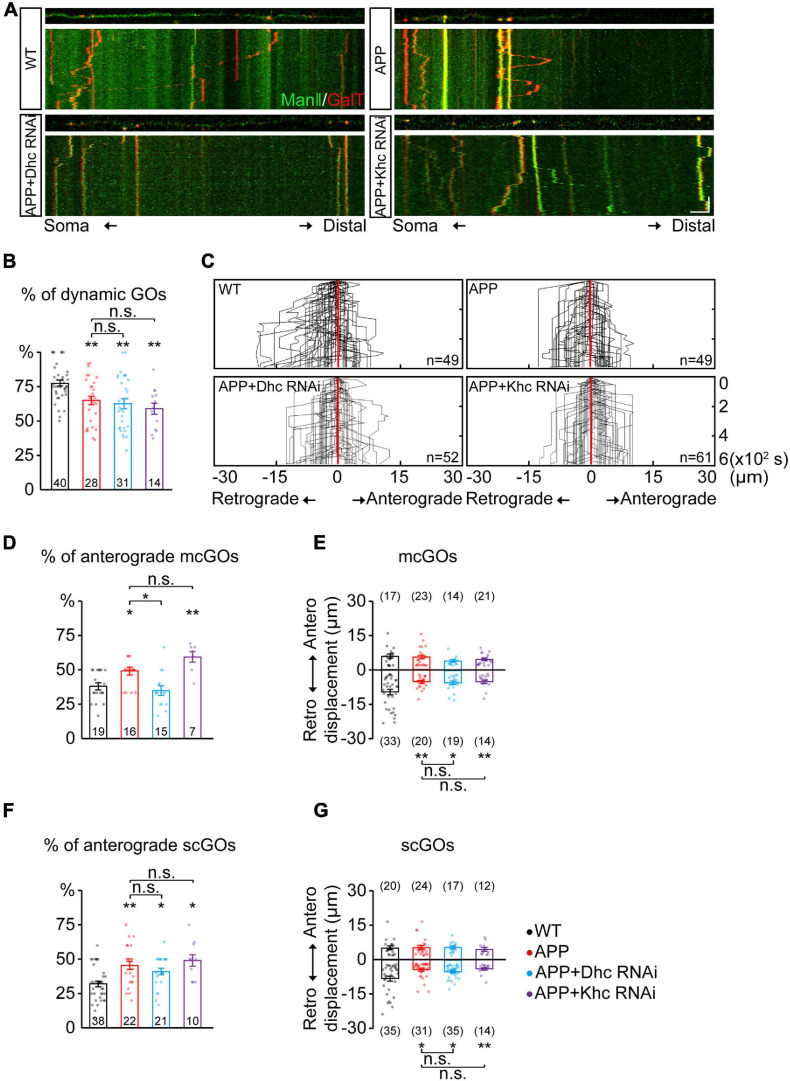
APP enhances the anterograde movements of mcGOs driven by *Dhc*. **(A)** Examples of the trajectories of dynamic GOs in the wild-type and APP neurons with RNAi knockdown of dynein heavy chain (*Dhc*) or kinesin heavy chain (*Khc*). *Top*: snapshots of the GOs in the straightened dendrites. *Bottom*: kymograph of time-lapse imaging of GOs for 10 min. Scale bar: 5 μm/2 min. **(B)** Quantification of the proportion of dynamic GOs. **(C)** Compound tracks of dynamic GOs. **(D,E)** Quantification of the features of dynamic mcGOs. **(D)** The percentage of anterograde movements, **(E)** displacements. **(F,G)** Quantification of the features of dynamic scGOs. **(F)** The percentage of anterograde movements; **(G)** displacements. Statistical significance was assessed with ANOVA tests (**P < 0.01; *P < 0.05; n.s., no significance).

**FIGURE 4:**
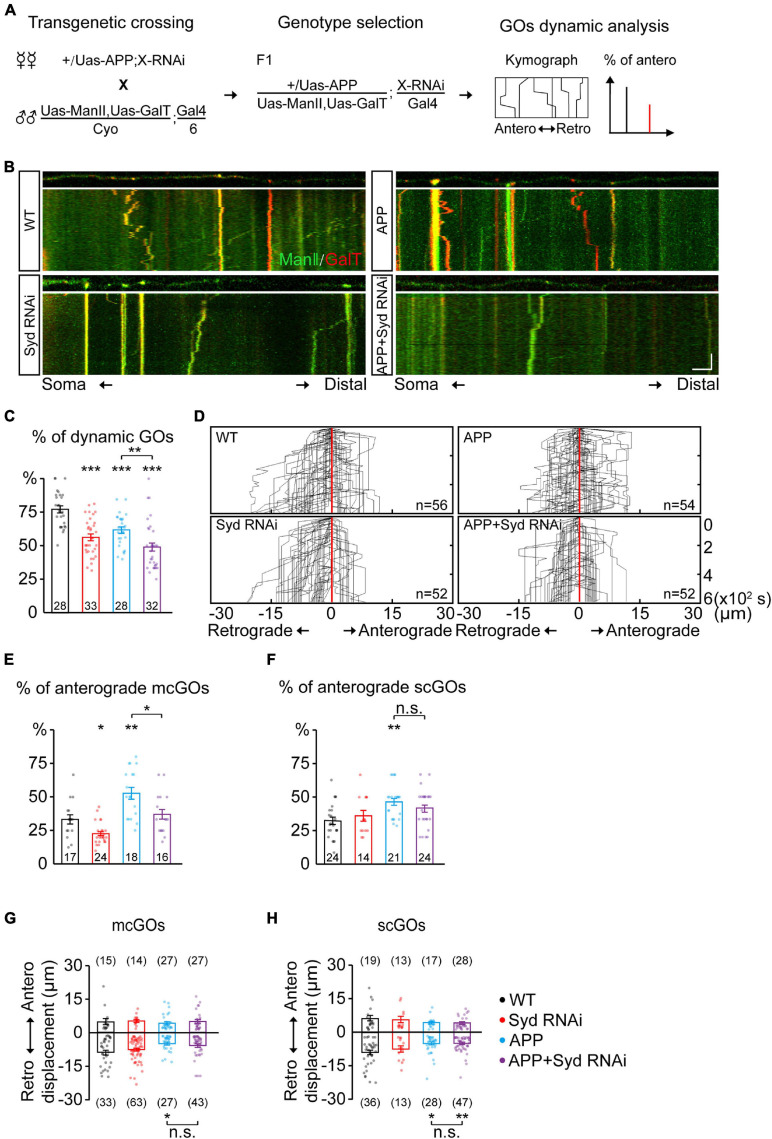
Syd is required for the alteration of mcGO dynamics induced by APP. **(A)** Flow of loss-of-function screening of candidate adaptor proteins which may be involved in the GO dynamics. **(B)** Examples of the trajectories of GO movements in the wild-type and APP neurons with *Syd* knockdown. *Top*: snapshots of the GOs in the straightened dendrites. *Bottom*: kymograph of time-lapse imaging of GOs for 10 min. Scale bar: 5 μm/2 min. **(C)** Quantification of the proportion of dynamic GOs. **(D)** Compound tracks of dynamic GOs. **(E,F)** Quantification of the percentage of anterograde movements for **(E)** mcGOs, and **(F)** scGOs. **(G,H)** Quantification of the displacements for **(G)** mcGOs and **(H)** scGOs. Statistical significance was assessed with ANOVA tests (***P < 0.001; **P < 0.01; *P < 0.05; n.s., no significance).

### Statistical Analysis

Data were analyzed using a two-tailed Student’s *t*-test for two groups comparisons, and ANOVA tests for multiple groups comparisons. Given that the variances of the groups were not homogeneous, the Brown–Forsythe and Welch ANOVA tests with *post hoc* comparisons *via* Tamhane’s T2 test were performed. Significance levels were presented as follows: ^∗∗∗^*P* < 0.001; ^∗∗^*P* < 0.01; ^∗^*P* < 0.05; n.s., no significance. Data were presented as mean ± SEM.

## Results

### APP Alters the Distribution of mcGOs in the Dendrites

To investigate the APP regulation of GOs, we first examined the correlation between APP and GOs by a colocalization analysis. Using fluorescent protein-tagged APP (APP-GFP) and GalT (GalT-TagRFPt, a *trans*-Golgi marker), we observed the location of APP and GOs in the dendrites of C3da neurons, a classic model for dendrite development (Grueber et al., 2002). APP presented as punctate patterns in both the soma and dendrites, and colocalized with the somal Golgi apparatus and dendritic GOs (Figure 1A). Quantification of the overlap between GalT and APP showed that it accounted for about 84.6% of GalT (Figure 1B) and 49.6% of APP in the dendrites (Figure 1C). Similar results were also obtained for the overlap between APP and ManII (ManII-TagRFPt, a *medial*-Golgi marker) (Figures 1A–C). Meanwhile, *in vivo* time-lapse imaging showed that both GOs and APP were dynamic in the dendrites (Figure 1D). Notably, the proportion of mobile GOs was decreased in the APP-colocalized GOs when compared to the puncta representing the lone GOs (Figure 1E). There was no significant difference between the lone GOs and GOs in the wild-type neurons (Figure 1E). These results indicate that APP resides in the vast majority of GOs, and alters the movements of GOs.

We next examined the distribution of GOs in the dendrites (Figure 1F). Considering the existence of both stacked mcGOs and disconnected scGOs in the dendrites (Zhou et al., 2014), the fluorescently tagged *medial*- and *trans*-Golgi markers (ManII-GFP and GalT-TagRFPt) were used simultaneously. To quantify the distribution of GOs, the dendrites were divided into three segments according to the distance to the soma: proximal (0–100 μm), medial (100–200 μm), and distal (>200 μm). The results showed that the distribution of mcGOs had a pronounced shift between the wild-type neurons and those which overexpressed human APP with Swedish mutation (APP neurons): the mcGOs were enriched in the proximal part of the dendrite in the APP neurons, whereas they were dominant in the distal part of the dendrite in the wild-type neurons (Figure 1G). However, the distribution of scGOs showed no change in the APP neurons (Figure 1H). These findings indicate that APP shifts the distribution tendency of mcGOs, from being enriched distally to proximally in the dendrites.

In summary, we found that the overwhelming majority of GOs had a resident APP, and this residence not only altered GO movements but also reversed the distribution of mcGOs.

### Dhc and Khc Cooperatively Maintain the mcGO Movements Equilibrium

Given that the distribution of mcGOs is related to the direction of their movements, the driving mechanism of mcGO movements was studied. Here, we utilized a pipeline to show the trajectories of GO movements (Figure 2A). It included three steps: time-lapse imaging, straightening of dendrites, and finally generation and analysis of a kymograph of GO movements. *In vivo* imaging showed that both mcGOs and scGOs can move bi-directionally (anterograde and retrograde) in the dendrites (Figures 2A,B). Motor proteins, including kinesin and dynein, have been reported to be involved in GO transport (Zheng et al., 2008; Kelliher et al., 2018). To reveal the motor proteins that control the mcGO dynamics, 10 candidate subunits of motor proteins were tested using RNAi (Supplementary Table 1). The results indicated that three RNAi lines led to a decrease in the proportion of dynamic GOs, which came from *Dhc* (Dhc-64c, dynein heavy chain subunit), *Khc* (kinesin heavy chain subunit), and, *Klc* (kinesin light chain subunit) (Supplementary Figure 1A). Among them, *Dhc* and *Khc* were found to regulate the direction of dynamic GOs (Supplementary Figure 1B and Figure 2C). Loss of *Dhc* and *Khc* had opposite effects on the direction of mcGO movements, with *Dhc* RNAi decreasing the proportion of anterograde movements of mcGOs but *Khc* RNAi increasing it (Figure 2D). These results indicate that *Dhc* is responsible for the anterograde movements of mcGOs, while *Khc* mediates the retrograde movements. Furthermore, the results also showed that the direction of scGO movements was only regulated by *Khc* RNAi rather than *Dhc* RNAi (Figure 2E). Neither *Dhc* RNAi nor *Khc* RNAi affected the displacement of the mcGO or scGO movements (Figures 2F,G). Taken together, these results suggest a model for the bidirectional movements of mcGOs: the anterograde and retrograde movements of mcGOs are driven by *Dhc* and *Kh*c, respectively, and the cooperation of *Dhc* and *Khc* maintains the equilibrium of the direction of the mcGO movements.

### APP Enhances the Anterograde Movements of mcGOs Driven by Dhc

Furthermore, the dynamic behaviors of GOs in the APP neurons were analyzed. APP also led to a decrease in the percentage of dynamic GOs (Figures 3A,B), similar to the knockdown of *Dhc* and *Khc*. The anterograde and retrograde movements of GOs in the APP neurons were quantified (Figure 3C). Results showed that the anterograde movements of mcGOs were increased in the APP neurons (Figure 3D). Meanwhile, the displacements of retrograde movements of mcGOs in the APP neurons became shorter when compared to the wild-type neurons, although the displacements of the anterograde movements remained unchanged (Figure 3E). Taken together, these results indicate that APP alters the direction and displacement of mcGO movements. In addition, the results also showed that the dynamic behaviors of scGOs in the APP neurons changed in a fashion similar to those of mcGOs (Figures 3F,G).

Furthermore, to explore the contributions of *Dhc* and *Khc* to the APP-induced alterations of GO movements, we examined the dynamic behaviors of GOs in the APP neurons after the knockdown of *Dhc* and *Khc*. The results showed that the proportion of the anterograde movements of mcGOs in the APP neurons with *Dhc* RNAi had no significant difference from those of the wild-type neurons, unlike those with *Khc* RNAi (Figure 3D). This demonstrated that the loss of *Dhc*, but not *Khc*, rescued the abnormal anterograde movements of mcGOs in the APP neurons. However, they did not restore the displacement of mcGO movements (Figure 3E). Neither *Dhc* RNAi nor *Khc* RNAi could recover the alterations of scGO movements induced by APP (Figures 3F,G).

Taken together, our results suggest that the anterograde movements of mcGOs are enhanced in the APP neurons, and this alteration can be recovered *via* the loss of *Dhc* but not *Khc*.

### Sunday Driver Specifically Mediates the Abnormality of the mcGO Movement Direction Induced by APP

The adaptor proteins act as bridges for motor proteins to recognize and anchor specific cargo (Karcher et al., 2002). To identify the adaptor proteins associated with mcGOs, we performed a loss-of-function screening for 24 adaptor proteins that had been reported to be involved in cargo transport (Supplementary Table 2 and Figure 4A). By comparing the motility of GOs, we found five proteins which induced a decrease in the proportion of dynamic GOs when knocked down (Supplementary Figure 2A). These proteins were Htt, Lva, Miro, NudE, and Syd. Next, we examined the direction of GO movements. Only Syd, Lva, and NudE played a role in it (Supplementary Figure 2B and Figures 4B,D). Syd was specific to mcGOs: *Syd* RNAi led to a decrease in the proportion of the anterograde movements of mcGOs, but not scGOs (Figures 4E,F). Lva and NudE regulated both mcGOs and scGOs. The difference between the function of Lva and NudE was that *Lva*^*DN*^ decreased the proportion of the anterograde movements, while *NudE* RNAi increased it (Supplementary Figures 3A–C).

Furthermore, we tested the function of these three proteins in the APP neurons by examining three characteristics of the GO movements: the motility of GOs and the direction and displacement of dynamic GOs. The results showed that, for mcGOs, *Syd* RNAi restored the percentage of the anterograde movements in the APP neurons to normal (Figure 4E), while the loss-of-function of *Lva* and *NudE* did not (Supplementary Figure 3B). Meanwhile, the motility of GOs and the displacements of mcGO movements were not recovered, nor were the characteristics of scGO movements (Figures 4C,F–H and Supplementary Figures 3C–E). These results suggest that the APP-induced alteration of the direction of mcGO movements is specifically mediated by Syd, not Lva and NudE, although both Lva and NudE are also involved in the regulation of the direction of mcGO movements.

In addition, we checked the localization of Syd using the fluorescent protein-tagged Syd (Syd-EGFP). It showed that Syd presented as the puncta in C3da neurons and colocalized with both somal Golgi and dendritic GOs (Supplementary Figures 4A,B). Then, by using the fluorescence signal of Syd-EGFP in soma, we evaluated the specificity of *Syd* RNAi. The average fluorescence intensity of Syd-EGFP was significantly reduced when it was expressed with *Syd* RNAi, whereas it was slightly reduced in the control RNAi (*Veli* RNAi, which was found to have no effect on the GO movements as shown in Supplementary Figure 2). Furthermore, the endogenous fluorescence signals from anti-HRP remained stable in all three groups (Supplementary Figures 5A–D). These results indicate that Syd was specifically knocked down by *Syd* RNAi.

Taken together, our findings demonstrate that Syd specifically mediates the anterograde movements of mcGOs, and is also required for APP’s regulation of this process.

### Syd Contributes to Dendritic Branching Defects Induced by APP

**FIGURE 5:**
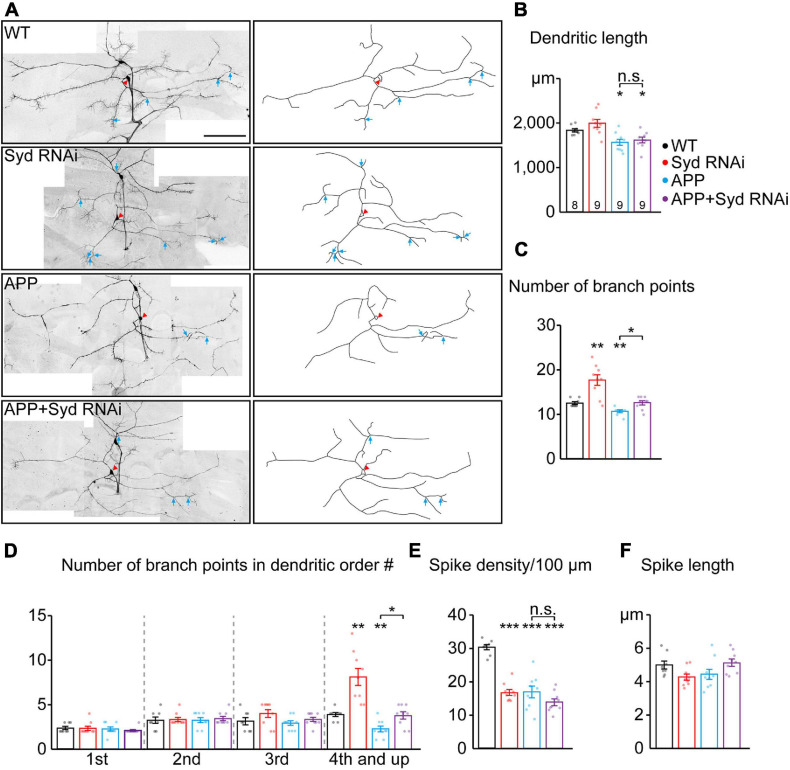
Loss of *Syd* recovers the dendritic defects induced by APP. **(A)** The dendritic morphologies of C3da ddaA neurons in the wild-type and APP neurons following the loss of *Syd*. Raw images (*left*) and tracings of dendritic branches (*right*, the numerous F-actin-based “dendritic spikes” of C3da neurons are not included). The red arrowhead points to the soma and blue arrows to the branch points of the fourth order and up. Scale bar: 100 μm. **(B–F)** Quantification of dendritic morphologies. **(B)** The total dendrite length, **(C)** the number of total branch points, **(D)** the number of branch points in different orders; **(E,F)** the characteristics of dendritic spikes: **(E)** density/100 μm, **(F)** average length. Statistical significance was assessed with ANOVA tests (***P < 0.001; **P < 0.01; *P < 0.05; n.s., no significance).

Dendritic defects are one of the pathological features of AD (Baloyannis, 2009). It has been reported that GOs are involved in dendrite development and morphogenesis (Horton et al., 2005; Ye et al., 2007; Ori-McKenney et al., 2012). Given the involvement of Syd in the APP-induced abnormal GO dynamics, the role of Syd in APP-induced dendritic defects was explored. We first characterized the dendritic morphology of C3da ddaA neurons with the loss of *Syd* (Figures 5A–F). The results showed that Syd regulated both the features of the dendrites and dendritic spikes. Compared with the wild-type, *Syd* RNAi led to an increase in the total number of dendritic branches, especially in the high-order branch points (fourth-order and up; Figures 5C,D), and a significant decrease in the density of dendritic spikes (Figure 5E). Meanwhile, the length of both dendrites and dendritic spikes showed no difference between the *Syd* RNAi and wild-type neurons (Figures 5B,F). These results suggest that Syd is necessary for the maintenance of a normal dendritic morphology.

Next, we examined the dendritic morphology of the APP neurons after *Syd* knockdown. The results showed that the absence of Syd partially rescued the dendrite phenotypes in the APP neurons without affecting the characteristics of the dendritic spikes. Consistent with the previously reported dendritic pathology in AD studies (Spires et al., 2005; Alpár et al., 2006), APP neurons showed a significant decrease in the dendrite length, the total number of dendritic branch points, in particular, high-order arbors, and the density of dendritic spikes (Figures 5B–E). *Syd* RNAi could restore the number of dendritic branches in the APP neurons to normal, especially the number of high-order dendritic branches (Figures 5C,D). However, *Syd* RNAi failed to rescue the total dendrite length (Figure 5B) and spike density in the APP neurons (Figure 5E).

Taken together, our findings indicate that the *Syd* RNAi and overexpressed APP have opposite regulatory effects on the complexity of dendritic arborization: *Syd* RNAi leads to an increase, while APP induces a decline. In particular, *Syd* RNAi can restore the dendritic branching to normal in the APP neurons. Concerning the density of dendritic spikes, the functions are the same: both APP and *Syd*-RNAi cause a decrease in the spike density, and *Syd*-RNAi cannot rescue this defect in the APP neurons. Syd does not have any effect on the alteration to the length of the dendrites and dendritic spikes induced by APP. Thus, these results suggest that Syd specifically mediates the APP-induced branching defects in the dendrites.

## Discussion

Here, we revealed that the dynein subunit Dhc and adaptor protein Syd mediate the APP’s regulation of the direction of mcGO movements. First, we found that APP exhibited colocalization and co-trafficking with GOs. In particular, APP reversed the distribution of mcGOs in the dendrites. Next, knockdown tests showed that the anterograde and retrograde movements of mcGOs are driven by Dhc and Khc, respectively, thus, suggesting a model of how the bidirectional movement of mcGOs are regulated. Furthermore, we found that Dhc, but not Khc, mediated the increased anterograde movements of mcGOs induced by APP. Finally, loss-of-function screening identified that Syd is required for APP to induce an alteration in the direction of the mcGO movements. Syd also was shown to mediate the dendritic branching defect induced by APP.

The abnormality of the direction of GO movements is thought to be a new feature of the APP-caused Golgi defects. Previous studies have demonstrated the correlation between APP and Golgi units in the dendrites. APP (Das et al., 2013) and its β-secretase, BACE-1 (Das et al., 2016), co-localize with the interior of Golgi-derived vesicles in the dendrites, and the α-secretase ADAM10 traffics from GOs to the synaptic membranes (Saraceno et al., 2014). Here, our results displayed a direct link between APP and GOs by showing their co-localization and co-trafficking in the dendrites. These indicate that GOs serve as the carriers of APP trafficking in the dendrites. Furthermore, the impact of APP residency on GOs was discussed. We pointed out that the alteration of the direction of mcGO movements is an important feature of APP’s regulation of GOs, which leads to a reversal of the distribution of mcGOs, and then, causes dendritic defects. These results indicate that APP not only impairs the somal Golgi apparatus, but also leads to defects of the dendritic GOs, pointing to a new direction for the study of Golgi defects in AD.

Our results reveal that Syd serves as a bridge for APP to alter mcGO movements. Several molecules have been studied in the regulation of GO transport and distribution: dominant-negative Lva (*Lva*^*DN*^) changes the distribution of GOs (Ye et al., 2007), and the localization of NudE at GOs inhibits their anterograde movements (Arthur et al., 2015). Here, we found a new regulator of GO transport, Syd, which is the homolog of the mammalian JIP3 in *Drosophila*. JIP3/Syd is involved in the axonal organelle transport, such as that of lysosomes (Gowrishankar et al., 2017) and early endosomes (Abe et al., 2009), and defects of the JIP3-dependent axonal lysosome transport aggravate Aβ accumulation in an AD model (Gowrishankar et al., 2017). Here, we discovered that Syd localizes at GOs in the dendrites. Furthermore, Syd specifically participates in the transport of mcGOs: *Syd* RNAi suppresses the anterograde movements of mcGOs, but not scGOs. This demonstrates that, consistent with Dhc, Syd mediates the anterograde movements of mcGOs, suggesting that Syd is an adaptor protein between Dhc and mcGOs. Furthermore, we discovered that *Syd* RNAi restores the change in mcGO movement direction induced by APP, whereas neither *Lva*^*DN*^ nor *NudE* RNAi do so. In summary, we propose that Syd, as the adaptor protein of mcGO movements, specifically mediates APP’s regulation of the direction of mcGO movements.

Our results also demonstrate the functional differences between mcGOs and scGOs. After these two types of compartmental organization of GOs in dendrites were first reported (Zhou et al., 2014), Valenzuela and Perez (2015) put forward the hypothesis that mcGOs and scGOs play different roles in the secretory pathway: mcGOs are responsible for the long-distance transport of proteins to dendritic branch points and synaptic junctions, while scGOs deliver the proteins to their destinations, thereby promoting local protein transport to the membrane. Thus, it is speculated that the functional differences between mcGOs and scGOs are one of the reasons for the different distribution changes. Moreover, the results from the loss-of-function of Dhc and Khc pointed out that the mechanisms underlying mcGO and scGO movements are different. Both Dhc and Khc can regulate the direction of mcGO movements, while that of scGOs can only be regulated by Khc. This distinction was also confirmed by the fact that Syd only regulates the direction of mcGO movements, but not that of scGOs. Moreover, we also found that mcGOs, but not scGOs, play important roles in neuronal development: only mcGOs regulate dendritic branching through Syd.

GO defects could be previously unrecognized features of AD. Dendritic pathology occurs in the early stages of other neurodegenerative disorders (Baloyannis, 2009). Recently, dendritic pathology related to GOs has been studied in a variety of neurodegenerative diseases: *LRRK2* mutation, one of the pathogenic factors in Parkinson’s disease, suppresses dendrite arborization by regulating GO dynamics (Lin et al., 2015); also, nuclear polyglutamine (polyQ), a putative factor in Machado-Joseph’s disease, induces a defective terminal dendrite elongation by impairing GOs synthesis (Chung et al., 2017). In AD, we found that *Syd* RNAi not only restores the abnormality of mcGO movement direction induced by APP, but also rescues the defect of dendritic branching. These imply that abnormal mcGO dynamics contribute to the dendritic defects, thereby suggesting that GO defects can be considered as indicators of an early stage of AD.

The present work also indicates that APP’s regulation of GO dynamics is reflected in a number of ways: APP alters the characteristics of both mcGO and scGO movements, including motility, direction, and displacement. Here, we only reveal the regulatory mechanism affecting the direction of mcGO movements. These results suggest that the regulation of GO movements by APP is achieved through multiple routes. The mechanism underlying the regulation of the motility and displacement of GO movements requires further investigation, which will help to reveal the role of GOs deficiencies in AD.

## Data Availability Statement

The raw data supporting the conclusions of this article will be made available by the authors, without undue reservation.

## Author Contributions

WZ and JC conceived this project. WZ, JC, and QD designed the experiments. QD conducted the experiments and analyzed the data. GC carried out the molecular experiments. WZ, JC, YZ, and QD wrote the manuscript. All authors contributed to the article and approved the submitted version.

## Conflict of Interest

The authors declare that the research was conducted in the absence of any commercial or financial relationships that could be construed as a potential conflict of interest.
