# Supplementary material for: Sunday Driver Mediates Multi-Compartment Golgi Outposts Defects Induced by Amyloid Precursor Protein
Source: Front Neurosci. 2021 Jun 1;15:673684. doi: 10.3389/fnins.2021.673684 (PMC8205063; doi:10.3389/fnins.2021.673684)
Supplement: Supplementary file 1 [file Image_1.pdf]

Supplementary Material

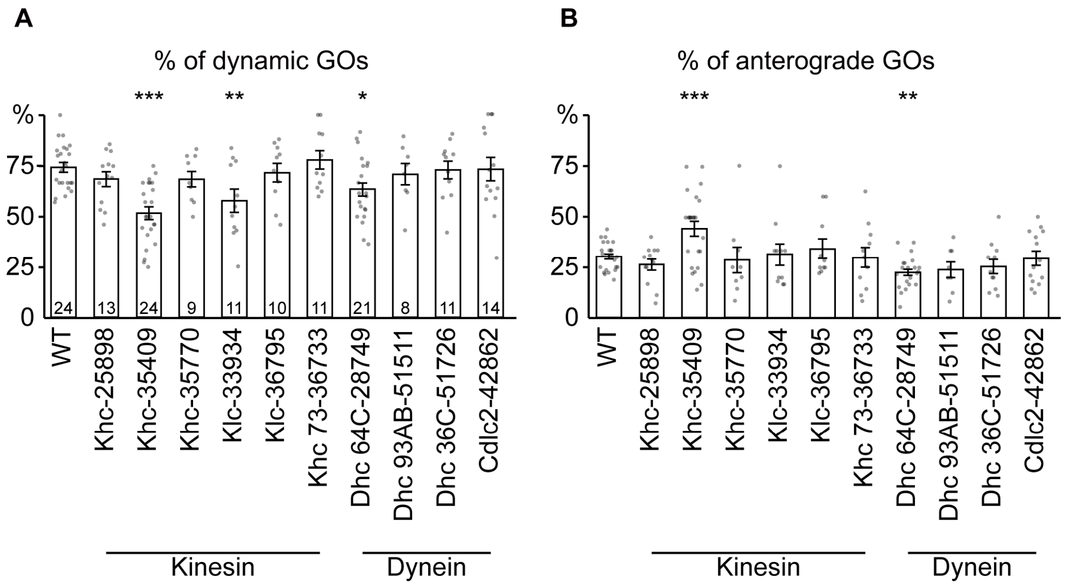

Supplementary Figure 1. RNAi-based knockdown tests identifying motor protein subunits that regulate GO dynamics. (A) The proportion of dynamic GOs. **(B)** The percentage of anterograde GO movements. Statistical significance was assessed with Student's *t*-test (****P* < 0.001; ***P* < 0.01; **P* < 0.05).

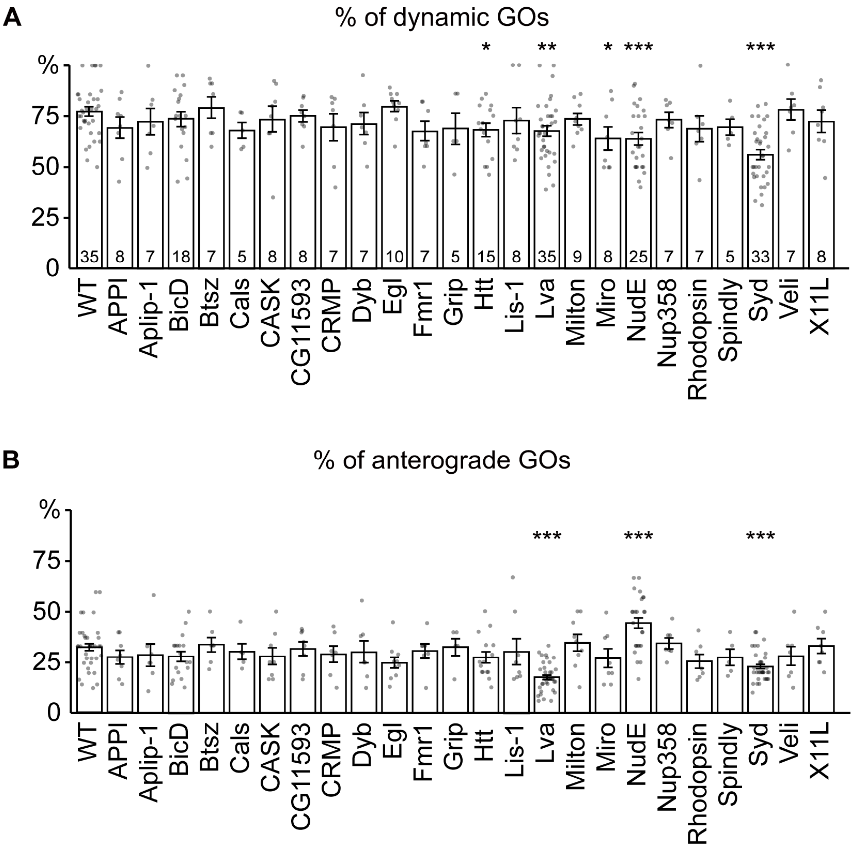

Supplementary Figure 2. Screening results of the candidate adaptor proteins involved in the regulation of GO dynamics. (A) Quantification of the proportion of dynamic GOs. **(B)** The percentage of anterograde GO movements. Statistical significance was assessed with Student's *t*-test (***P < 0.001; **P < 0.01; *P < 0.05).

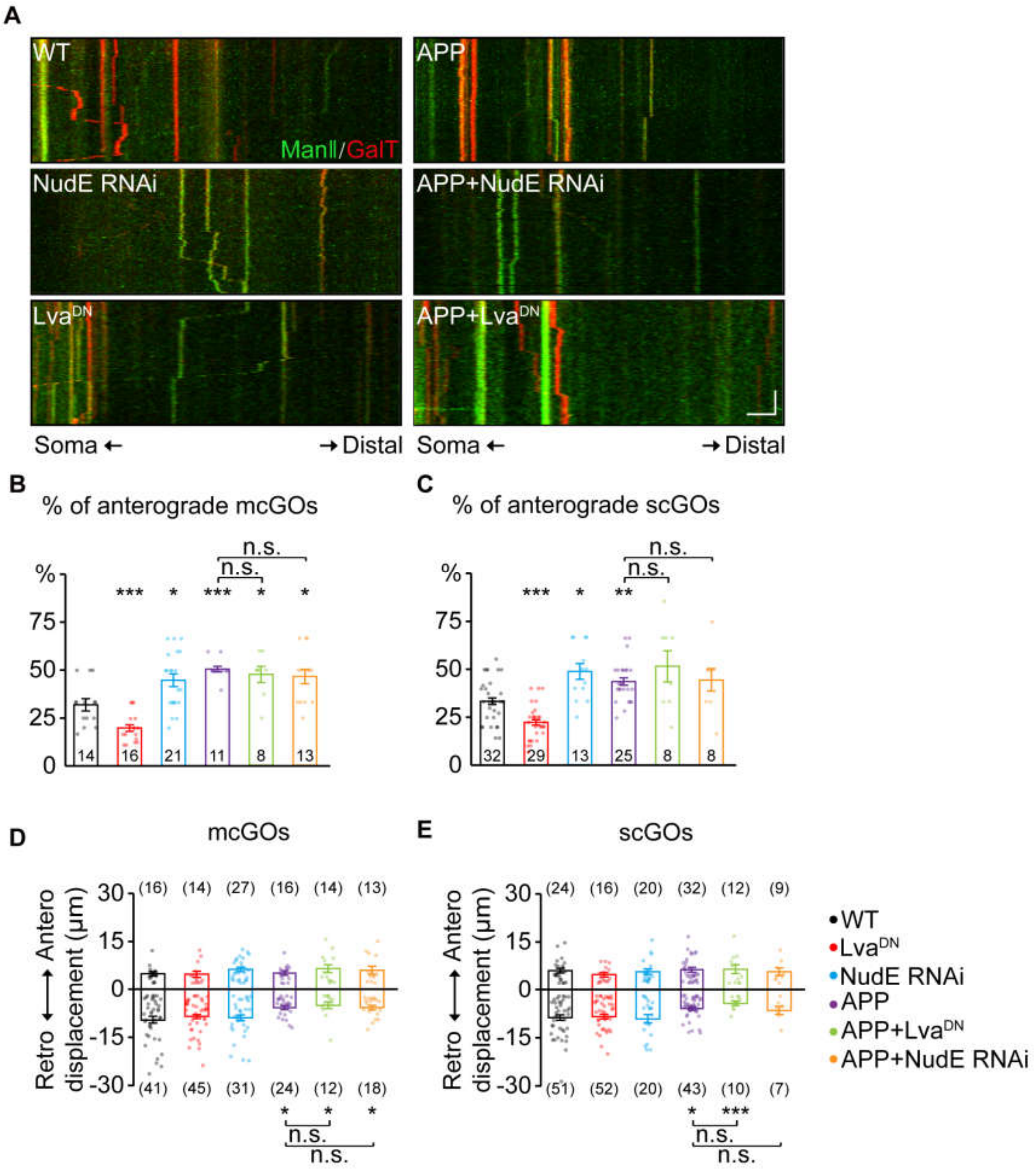

Supplementary Figure 3. Neither *Lva^{DN}* nor *NudE* RNAi rescue the changes of GO dynamics induced by APP. (A) kymograph showing the GO movements in wild-type and APP neurons with *Lva^{DN}* or *NudE* RNAi. Scale bar: 5 μm / 2 min. **(B-C)** Quantification of the percentage of anterograde movements for **(B)** mcGOs, and **(C)** scGOs. **(D-E)** Quantification of the displacements for **(D)** mcGOs, and **(E)** scGOs. Statistical significance was assessed with ANOVA tests (***P < 0.001; **P < 0.01; *P < 0.05; n.s., no significance).

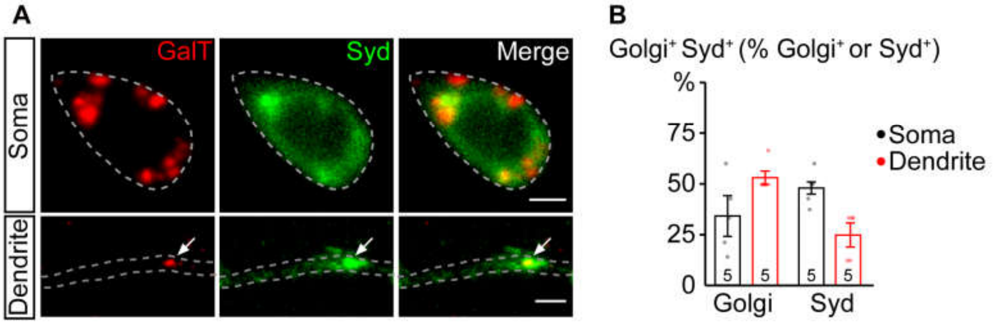

Supplementary Figure 4. Syd localizes at GOs in dendrites. (A) Colocalization between Syd (green, Syd-GFP) and Golgi complex (red, GalT-TagRFPt) in soma and dendrites. Scale bar: 2 µm. **(B)** Quantification of colocalization between Syd and Golgi complex in soma and dendrites as the percentage of total Golgi complex (Golgi) or Syd.

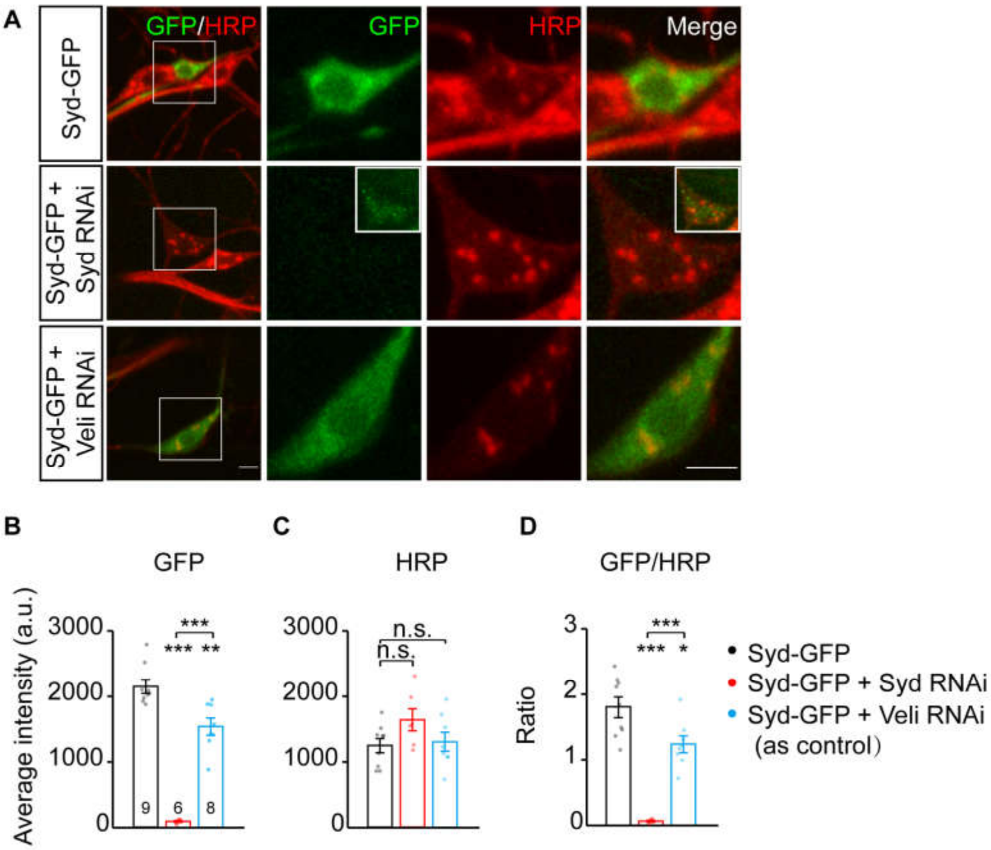

Supplementary Figure 5. Syd was specifically knocked down by *Syd* RNAi. (A) Example images of Syd-GFP in the soma. Neurons were labeled by Syd-GFP (green, as Syd marker) and anti-HRP (red, as the endogenous membrane protein marker). Upper panel: expressing Syd-GFP only; middle: Syd-GFP with *Syd* RNAi; lower: Syd-GFP with *Veli* RNAi (as control). The insets in the middle panel showed a weaker Syd-GFP signal when *Syd* RNAi was expressed, which increased the laser power to 12.5 times that of the control. (**B-C**) Quantification of average fluorescence intensity for (**B**) Syd-GFP and (**C**) HRP. (**D**) Ratio of the average fluorescence intensity of Syd-GFP and HRP. Statistical significance was assessed with ANOVA tests (***P < 0.001; **P < 0.01; *P < 0.05; n.s., no significance).

Table S1. Transgenic RNAi fly stocks of the subunits of motor proteins

Family	Gene Symbol	Gene Description	Stock Number
Kinesin	Khc	Kinesin heavy chain	BDSC#25898
			BDSC#35409
			BDSC#35770
	Klc	Kinesin light chain	BDSC#33934
			BDSC#36795
	Khc 73	Kinesin heavy chain 73	BDSC#36733
Dynein	Dhc 64C	Dynein heavy chain at 64C	BDSC#28749
	Dhc 93AB	Dynein heavy chain at 93AB	BDSC#51511
	Dhc 36C	Dynein heavy chain at 36C	BDSC#51726
	Cdlc2	Cytoplasmic dynein light chain 2	BDSC#42862

Table S2. Transgenic fly stocks used for loss-of-function screening of adaptor proteins

Gene Description	Gene Description	Reference	Stock Number
APP-like protein interacting protein 1	Aplip1	(Verhey et al. 2001, Horiuchi et al. 2005)	THU2164
β amyloid protein precursor-like	Appl	(Kamal et al. 2001)	THU3938
Bicaudal D	BicD	(Hoogenraad et al. 2001)	THU0296
Bitesize	Btsz	(Arimura et al. 2009)	THU4140
Calsyntenin-1	Calsyntenin-1	(Konecna et al. 2006)	THU2051
CASK		(Setou et al. 2000)	THU1046
CG11593		(Akamatsu et al. 2015)	THU1116
Collapsin Response Mediator Protein	CRMP	(Kimura et al. 2005, Arimura et al. 2009)	TH02545.N
Dystrobrevin	Dyb	(Macioce et al. 2003)	THU1127
Egalitarian	Egl	(Navarro et al. 2004)	THU0490
Fmr1		(Davidovic et al. 2007)	THU0740
Glutamate receptor interacting protein	Grip	(Setou et al. 2002)	THU5356
Huntingtin	Htt	(Caviston et al. 2007, Colin et al. 2008)	THU5643
Lissencephaly-1	Lis-1	(Sitaram et al. 2012)	THU3056
Lava lamp	Lva	(Papoulas et al. 2005)	BDSC#55055
Milton	Milt	(Glater et al. 2006, van Spronsen et al. 2013)	THU4715

To be continued

Mitochondrial Rho	Miro	(Glater, Megeath et al. 2006, Russo et al. 2009)	THU4782
NudE		(Arthur et al. 2015)	BDSC#41860
Nucleoporin 358Kd(Nup358)	Nup358	(Cho et al. 2007)	THU1196
Rhodopsin 6	Rh6	(Tai et al. 1999)	TH03512.N
Spindly		(Griffis et al. 2007)	THU1515
Sunday driver	Syd	(Bowman et al. 2000, Cavalli et al. 2005)	TH01110.N2
Veli		(Setou, Nakagawa et al. 2000)	THU3174
X11L		(Setou, Nakagawa et al. 2000)	THU2492

Reference

Akamatsu, R., N. Ishida-Kitagawa, T. Aoyama, C. Oka and M. Kawaichi (2015). "BNIP-2 binds phosphatidylserine, localizes to vesicles, and is transported by kinesin-1." Genes Cells **20**(2): 135-152. DOI: 10.1111/gtc.12209.

Arimura, N., A. Hattori, T. Kimura, S. Nakamuta, Y. Funahashi, S. Hirotsune, et al. (2009). "CRMP-2 directly binds to cytoplasmic dynein and interferes with its activity." J Neurochem **111**(2): 380-390. DOI: 10.1111/j.1471-4159.2009.06317.x.

Arimura, N., T. Kimura, S. Nakamuta, S. Taya, Y. Funahashi, A. Hattori, et al. (2009). "Anterograde transport of TrkB in axons is mediated by direct interaction with Slp1 and Rab27." Dev Cell **16**(5): 675-686. DOI: 10.1016/j.devcel.2009.03.005.

Arthur, A. L., S. Z. Yang, A. M. Abellaneda and J. Wildonger (2015). "Dendrite arborization requires the dynein cofactor NudE." J Cell Sci **128**(11): 2191-2201. DOI: 10.1242/jcs.170316.

Bowman, A. B., A. Kamal, B. W. Ritchings, A. V. Philp, M. McGrail, J. G. Gindhart, et al. (2000). "Kinesin-dependent axonal transport is mediated by the sunday driver (SYD) protein." Cell **103**(4): 583-594. DOI: 10.1016/s0092-8674(00)00162-8.

Cavalli, V., P. Kujala, J. Klumperman and L. S. Goldstein (2005). "Sunday Driver links axonal transport to damage signaling." J Cell Biol **168**(5): 775-787. DOI: 10.1083/jcb.200410136.

Caviston, J. P., J. L. Ross, S. M. Antony, M. Tokito and E. L. Holzbaur (2007). "Huntingtin facilitates dynein/dynactin-mediated vesicle transport." Proc Natl Acad Sci U S A **104**(24): 10045-10050. DOI: 10.1073/pnas.0610628104.

Cho, K. I., Y. Cai, H. Yi, A. Yeh, A. Aslanukov and P. A. Ferreira (2007). "Association of the kinesin-binding domain of RanBP2 to KIF5B and KIF5C determines mitochondria localization and function." Traffic **8**(12): 1722-1735. DOI: 10.1111/j.1600-0854.2007.00647.x.

Colin, E., D. Zala, G. Liot, H. Rangone, M. Borrell-Pagès, X. J. Li, et al. (2008). "Huntingtin

phosphorylation acts as a molecular switch for anterograde/retrograde transport in neurons." Embo j **27**(15): 2124-2134. DOI: 10.1038/emboj.2008.133.

Davidovic, L., X. H. Jaglin, A. M. Lepagnol-Bestel, S. Tremblay, M. Simonneau, B. Bardoni, et al. (2007). "The fragile X mental retardation protein is a molecular adaptor between the neurospecific KIF3C kinesin and dendritic RNA granules." Hum Mol Genet **16**(24): 3047-3058. DOI: 10.1093/hmg/ddm263.

Glater, E. E., L. J. Megeath, R. S. Stowers and T. L. Schwarz (2006). "Axonal transport of mitochondria requires milton to recruit kinesin heavy chain and is light chain independent." J Cell Biol **173**(4): 545-557. DOI: 10.1083/jcb.200601067.

Griffis, E. R., N. Stuurman and R. D. Vale (2007). "Spindly, a novel protein essential for silencing the spindle assembly checkpoint, recruits dynein to the kinetochore." J Cell Biol **177**(6): 1005-1015. DOI: 10.1083/jcb.200702062.

Hoogenraad, C. C., A. Akhmanova, S. A. Howell, B. R. Dortland, C. I. De Zeeuw, R. Willemsen, et al. (2001). "Mammalian Golgi-associated Bicaudal-D2 functions in the dynein-dynactin pathway by interacting with these complexes." Embo j **20**(15): 4041-4054. DOI: 10.1093/emboj/20.15.4041.

Horiuchi, D., R. V. Barkus, A. D. Pilling, A. Gassman and W. M. Saxton (2005). "APLIP1, a kinesin binding JIP-1/JNK scaffold protein, influences the axonal transport of both vesicles and mitochondria in *Drosophila*." Curr Biol **15**(23): 2137-2141. DOI: 10.1016/j.cub.2005.10.047.

Kamal, A., A. Almenar-Queralt, J. F. LeBlanc, E. A. Roberts and L. S. Goldstein (2001). "Kinesin-mediated axonal transport of a membrane compartment containing beta-secretase and presenilin-1 requires APP." Nature **414**(6864): 643-648. DOI: 10.1038/414643a.

Kimura, T., H. Watanabe, A. Iwamatsu and K. Kaibuchi (2005). "Tubulin and CRMP-2 complex is transported via Kinesin-1." J Neurochem **93**(6): 1371-1382. DOI: 10.1111/j.1471-4159.2005.03063.x.

Konecna, A., R. Frischknecht, J. Kinter, A. Ludwig, M. Steuble, V. Meskenaite, et al. (2006). "Calsyntenin-1 docks vesicular cargo to kinesin-1." Mol Biol Cell **17**(8): 3651-3663. DOI: 10.1091/mbc.e06-02-0112.

Macioce, P., G. Gambara, M. Bernassola, L. Gaddini, P. Torreri, G. Macchia, et al. (2003). "Beta-dystrobrevin interacts directly with kinesin heavy chain in brain." J Cell Sci **116**(Pt 23): 4847-4856. DOI: 10.1242/jcs.00805.

Navarro, C., H. Puthalakath, J. M. Adams, A. Strasser and R. Lehmann (2004). "Egalitarian binds dynein light chain to establish oocyte polarity and maintain oocyte fate." Nat Cell Biol **6**(5): 427-435. DOI: 10.1038/ncb1122.

Papoulas, O., T. S. Hays and J. C. Sisson (2005). "The golgin Lava lamp mediates dynein-based Golgi movements during *Drosophila* cellularization." Nat Cell Biol **7**(6): 612-618. DOI: 10.1038/ncb1264.

Russo, G. J., K. Louie, A. Wellington, G. T. Macleod, F. Hu, S. Panchumarthi, et al. (2009). "*Drosophila* Miro is required for both anterograde and retrograde axonal mitochondrial transport." J Neurosci **29**(17): 5443-5455. DOI: 10.1523/jneurosci.5417-08.2009.

Setou, M., T. Nakagawa, D. H. Seog and N. Hirokawa (2000). "Kinesin superfamily motor protein KIF17 and mLin-10 in NMDA receptor-containing vesicle transport." Science **288**(5472): 1796-1802. DOI: 10.1126/science.288.5472.1796.

Setou, M., D. H. Seog, Y. Tanaka, Y. Kanai, Y. Takei, M. Kawagishi, et al. (2002). "Glutamate-receptor-interacting protein GRIP1 directly steers kinesin to dendrites." Nature **417**(6884): 83-87. DOI: 10.1038/nature743.

Sitaram, P., M. A. Anderson, J. N. Jodoin, E. Lee and L. A. Lee (2012). "Regulation of dynein

localization and centrosome positioning by Lis-1 and asunder during *Drosophila* spermatogenesis." Development **139**(16): 2945-2954. DOI: 10.1242/dev.077511.

Tai, A. W., J. Z. Chuang, C. Bode, U. Wolfrum and C. H. Sung (1999). "Rhodopsin's carboxy-terminal cytoplasmic tail acts as a membrane receptor for cytoplasmic dynein by binding to the dynein light chain Tctex-1." Cell **97**(7): 877-887. DOI: 10.1016/s0092-8674(00)80800-4.

van Spronsen, M., M. Mikhaylova, J. Lipka, M. A. Schlager, D. J. van den Heuvel, M. Kuijpers, et al. (2013). "TRAK/Milton motor-adaptor proteins steer mitochondrial trafficking to axons and dendrites." Neuron **77**(3): 485-502. DOI: 10.1016/j.neuron.2012.11.027.

Verhey, K. J., D. Meyer, R. Deehan, J. Blenis, B. J. Schnapp, T. A. Rapoport, et al. (2001). "Cargo of kinesin identified as JIP scaffolding proteins and associated signaling molecules." J Cell Biol **152**(5): 959-970. DOI: 10.1083/jcb.152.5.959.
